# Scalable Synthesis of PtAu Nanoalloy-Decorated Hydrogenated TiO_2_ for High-Efficiency Indoor Formaldehyde Photodegradation

**DOI:** 10.3390/nano15090683

**Published:** 2025-04-30

**Authors:** Hairui Cai, Benjamin Yang, Jie Hou, Ziqi Wang, Zhuo Li

**Affiliations:** 1 MOE Key Laboratory for Non-Equilibrium Synthesis and Modulation of Condensed Matter, Key Laboratory of Shaanxi for Advanced Materials and Mesoscopic Physics, State Key Laboratory for Mechanical Behavior of Materials, School of Physics, Xi’an Jiaotong University, No. 28 West Xianning Road, Xi’an 710049, China; sherlock_hou@stu.xjtu.edu.cn (J.H.); 154922@stu.xjtu.edu.cn (Z.W.); 2 Shanghai American School Pudong Campus, No. 1600 Lingbai Highway, Shanghai 201201, China; benjamin01pd2026@saschina.org; 3 School of Electrical Engineering, Xi’an Jiaotong University, No. 28 West Xianning Road, Xi’an 710049, China; lizhuo3007@stu.xjtu.edu.cn

**Keywords:** formaldehyde, hydrogenated P25, photocatalyst, PtAu nanoalloys

## Abstract

Formaldehyde, a pervasive indoor air pollutant posing significant health risks, has driven extensive research into advanced mitigation strategies to ensure safer living environments. Herein, this study presents a synthesis method for the large-scale production of hydrogenated TiO_2_ (P25) loaded with PtAu nanoalloys (P25(H)-PtAu), using a combination of ball milling and high-temperature annealing. Hydrogenation-induced defect-rich TiO_2_ efficiently improves visible light absorption, enhancing the utilization of visible light in photocatalytic reactions. Mechanochemical ball milling was employed to prepare ultrasmall PtAu nanoalloys with a size of 3.7 ± 0.1 nm, which were uniformly dispersed on the surface of P25(H). Density functional theory (DFT) results indicate that PtAu nanoalloys synergistically enhance charge separation via Schottky junctions and surface reaction kinetics by optimizing reactant adsorption. As a result, P25(H)-PtAu achieves industrially relevant formaldehyde removal efficiency (97.8%) under ambient light conditions while maintaining scalability (10 g batches). This work provides a scalable framework for developing manufacturable photocatalysts, with immediate applications in heating, ventilation and air conditioning systems, and air purifiers.

## 1. Introduction

Formaldehyde (FA, HCHO), a pervasive indoor air pollutant emitted from construction materials, furniture, and consumer products, poses significant health risks ranging from acute irritations (ocular, nasal, and respiratory) to severe chronic conditions including nasopharyngeal carcinoma, leukemia, and immune dysfunction [1,2,3]. Particularly concerning is the cumulative toxicity associated with prolonged low-concentration exposure, necessitating urgent development of effective abatement strategies for modern indoor air quality management. Photocatalytic oxidation using titanium dioxide (TiO_2_) has emerged as a promising solution due to its sustainable mineralization of formaldehyde into CO_2_ and H_2_O under ultraviolet (UV) irradiation [4,5,6]. The mechanism relies on UV-induced electron-hole pairs generating reactive oxygen species (ROS, •OH, •O_2_^−^) for pollutant degradation [7,8]. However, the practical implementation of TiO_2_ is limited by its wide bandgap (3.2 eV) to UV light (λ < 387 nm).

Recent advances address these challenges through dual modification strategies. Hydrogenation treatment creates black TiO_2_(H) with oxygen vacancies and Ti^3^+/Ti-H moieties [9], inducing visible light absorption (400–800 nm) through mid-gap states while maintaining crystallinity. Although this enhances formaldehyde degradation kinetics under ambient light, insufficient active sites and residual charge recombination limit quantum efficiency (<15%) [10]. Concurrently, noble metal nanoparticle decoration (Pt, Au, Ag) demonstrates multifunctional benefits [11,12]. It effectively promotes the separation of electron-hole pairs, reduces electron recombination, and facilitates the adsorption and activation of formaldehyde and oxygen molecules [13]. Additionally, introducing noble metals can improve the light absorption of semiconductors, particularly in the visible region, through local surface plasmon resonance (LSPR), further boosting their formaldehyde degradation performance [14,15,16].

The efficiency of ROS generation and utilization critically determines the reaction kinetics in photocatalytic formaldehyde degradation [17]. Monometallic catalysts (pure Pt or Au) exhibit inherent limitations in adsorbing and activating key reactants [18,19,20]. Recent studies demonstrate that alloy catalysts can promote •OH generation and utilization [19,20]. For instance, Liu reported that the PtAg alloy was supported on HZSM-5, in which electrons could be transferred from Pt to Ag, optimizing the pollutants’ adsorption energy and achieving 100% formaldehyde conversion [19]. Furthermore, Wang et al. demonstrated that microwave-synthesized PtNi/Al_2_O_3_ achieves almost 100% HCHO degradation compared to monometallic Pt/Al_2_O_3_ (57%), attributed to Ni-induced charge separation and alloy-mediated active site exposure for ROS utilization [20]. Despite these advancements, significant challenges persist in catalyst synthesis methodologies. Conventional noble metal deposition methods (e.g., chemical reduction, photo-deposition) face challenges in controlling particle size distribution and noble metal-substrate (TiO_2_) adhesion at industrial scales [21,22,23]. While advanced synthesis strategies such as microwave-assisted solvothermal synthesis and atomic layer deposition have shown promise in addressing these issues [20,24], their reliance on specialized precursors or extreme operational parameters (vacuum conditions, etc.) is incompatible with industrial scale. Therefore, there is an urgent need to develop an efficient and scalable synthesis method capable of producing uniformly dispersed alloy nanoparticle-loaded TiO_2_ catalysts at an industrial scale.

Herein, we develop a scalable two-step synthesis of P25(H)-PtAu nanocomposites combining hydrogenation and mechanochemical alloying. Hydrogenation generates defect-rich black TiO_2_(H) with optimized visible absorption, while subsequent ball milling with commercially available metal precursors (H_2_PtCl_6_, HAuCl_4_) followed by thermal annealing produces uniformly dispersed PtAu nanoalloys (3.7 ± 0.1 nm). This synergistic design achieves an extended visible light response through bandgap engineering and enhanced charge separation due to the lower Fermi energy in PtAu. Furthermore, PtAu nanoalloys act as active sites for photocatalytic reactions, facilitating adsorption and activation of O_2_, H_2_O, and formaldehyde. The resulting TiO_2_(H)-PtAu photocatalysts showed a superior formaldehyde removal performance compared to commercial catalysts. Crucially, this methodology enables single-batch production of 10 g quantities with <5% performance variation, establishing a viable pathway for industrial-scale air purification applications.

## 2. Experimental Section

### 2.1. Chemicals and Materials

P25 (P25 titanium dioxide) was purchased from Evonik Degussa (Frankfurt, Germany). Key characteristics include a primary particle size of ~21 nm, a Brunauer–Emmett–Teller (BET) surface area of 50 ± 15 m^2^/g, a chemical purity of 99.5%, and a compaction density of ~130 g/L. Chloroplatinic acid hexahydrate (H_2_PtCl_6_·6H_2_O) and Trichlorogold hydrochloride hydrate (HAuCl_4_·3H_2_O), with a purity of 99.9%, were supplied by Shanghai Aladdin Biochemical Technology Co., Ltd. (Shanghai, China) for the synthesis of platinum and gold nanoparticles. Ethanol (C_2_H_6_O, 99.5%) and formaldehyde solution (aqueous solution containing 38% formaldehyde, FA, HCHO) were purchased from Sinopharm Chemical Reagent Co., Ltd. (Shanghai, China). The commercial product used in this study is “Museng”, which is explicitly labeled as containing anatase TiO_2_ and platinum (Pt) nanoparticles as its active components (Xi’an, China). No additional purification processes were applied to these chemicals before their use in the experiments. Deionized water was consistently utilized as the solvent throughout the experimental procedures.

### 2.2. Synthesis of Hydrogenated P25 Covered with PtAu Nanoparticles

**Figure 1:**
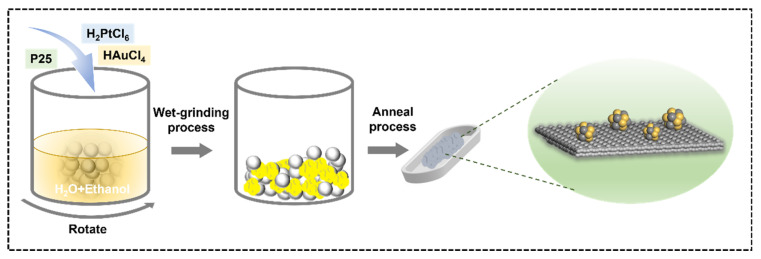
Schematic diagram of P25(H)-PtAu preparation.

To obtain hydrogenated TiO_2_ covered with PtAu alloy (abbreviated as P25(H)-PtAu), 3 g of P25, 1 mL of deionized H_2_O, 1 mL of ethanol and quantitative H_2_PtCl_6_·6H_2_O and HAuCl_4_·3H_2_O were added in an agate ball mill tank to form a sticky slurry. The mass ratio of P25, Pt, and Au was 100:2.5:2.5. The sticky slurry was ground by a wet-grinding process using ceramic milling balls (Φ = 5/8 mm) until the mixture was completely dry to form a yellow powder, with a rotation speed of 500 rpm. Then, the obtained yellow powder was put into an alumina crucible and annealed in 5% H_2_ in Ar at 500 °C for 2 h in a tube furnace. During this thermal treatment process, Pt^4+^ and Au^3+^ species were reduced to their metallic states through the thermal decomposition of the precursor salts, accompanied by a distinct color transformation of the powder from yellow to dark gray. After cooling down, the final product was washed thoroughly with distilled water and ethanol, and then dried at 60 °C for 5 h in a vacuum oven. P25(H)-PtAu preparation for 10 g batches can be performed by mechanical ball milling, as shown in Figure 1.

### 2.3. Characterization

The nanometer-ranged microstructure and element distribution of synthesized particles were analyzed using a Thermo Fisher Scientific Helios 5 (Waltham, MA, USA) scanning electron microscope (SEM). Atomic resolution scanning transmission electron microscopy (STEM) and energy-dispersive X-ray spectroscopy (EDS) were acquired using a double aberration-corrected Thermo Scientific Spectra 300 transmission electron microscope (Waltham, MA, USA) with a spatial resolution of 50 pm and a 300 kV accelerating voltage. The X-ray diffraction (XRD) measurements were applied to find out the sample crystal structure by a Bruker D8 ADVANCE diffractometer (Waltham, MA, USA) with a scan range of 20–90°. The UV–Vis diffuse reflectance absorption spectra (DRS) of catalysts were measured using a HITACHI U4100 instrument (Tokyo, Japan) with BaSO_4_ as the reference, and a wavelength range of 300–800 nm. X-ray photoelectron spectroscopy (XPS) measurements were conducted on a Thermo Fisher ESCALAB Xi^+^ (Waltham, MA, USA) with monochromatic Al Kα radiation (hν = 1486.69 eV) and with the pressure of the sample analysis chamber under high vacuum < 5 × 10^−10^ mbar. All binding energies were referenced to the C 1 s peak at 284.8 eV. Raman spectra were conducted on a Horiba Jobin-Yvon HR800 system (Pairs, France) with a 532 nm laser wavelength. Formaldehyde concentration testing was performed on a model P1 formaldehyde tester from Qingdao Miyu Technology Co., Ltd. (Qingdao, China) with an electrochemical sensor principle and a resolution of 0.01 mg/m^3^.

### 2.4. Formaldehyde Removal Measurements

The preparation of the catalyst film was as follows: The catalyst ink was prepared by mechanical ball milling. Specifically, 1 g of P25(H)-PtAu powder, 20 g of ceramic balls, and 20 mL of deionized water were added to a 100 mL Teflon ball mill tank. The mixture underwent ball milling at 500 rpm for 2 h under ambient conditions, achieving homogeneous dispersion through continuous mechanical impact. Subsequently, the colloidal suspension was diluted with deionized water to obtain a catalyst ink with a concentration of 2 mg/mL. This catalyst ink was then uniformly deposited onto acid-washed A4 cellulose paper using an airbrush coating system. The coated substrate underwent drying for 2 h until completely dry, obtaining a catalyst film.

**Figure 2:**
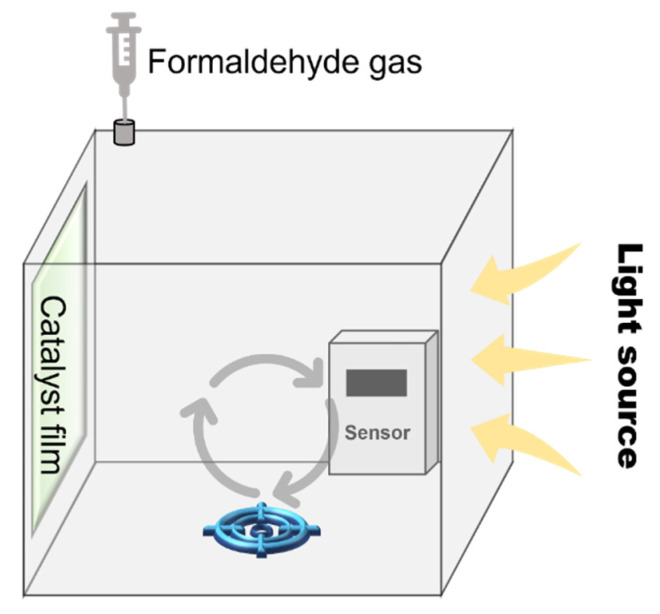
Schematic diagram of formaldehyde removal test set-up.

The performance assessment system was as follows: The evaluation platform comprised a transparent sealed chamber (dimension 40 cm × 27 cm × 26 cm) equipped with an FA sensor and a closed-loop gas circulation device (Figure 2). The catalyst film was vertically mounted on the chamber and faced to the outside light source (300 W xenon lamp, 50 mW/cm^2^). Before the test, 40 µL of FA solution was volatilized in a 125 mL sealed conical flask at 45 °C to obtain a fixed concentration of FA gas. At the beginning of the test, 10 mL of FA gas was injected into the chamber, and the gas circulation device and FA sensor were turned on immediately. The chamber was left in the dark for 10 min to establish the adsorption-desorption FA equilibrium state. Then, the light source was turned on to monitor the change in the FA vapor concentration as a function of light irradiation time using an online FA sensor placed inside the chamber. The efficiency of FA removal (%) was calculated as [1 − (C_t_/C_0_)] × 100, where C_0_ and C_t_ represent initial and time-dependent FA concentrations, respectively. In the photocatalytic stability test, after each photocatalytic cycle, the catalyst was carefully retrieved and subjected to a thermal regeneration protocol. Specifically, the catalyst film was placed in a blast oven at 80 °C for 2 h under continuous airflow, which can accelerate the removal of adsorbed organic intermediates.

### 2.5. DFT Calculations

The theoretical calculations were conducted using Vienna ab initio simulation packages (VASP). The projector augmented wave (PAW) pseudopotentials were used to describe the core and valence. The generalized gradient approximation (GGA) was performed with the Perdew–Burke–Ernzerhof (PBE) for the adopted exchange correlation potential. The cutoff energy of the plane wave basis set was fixed at 450 eV. A 3 × 3 × 1 Monkhorst–Pack K-point grid was used for geometric optimization. The forces on each atom were less than 0.01 eV/Å. The convergence criterion for energy is 10^−5^ eV. A vacuum space of 15 Å was used in the calculation to avoid interaction between the interfaces. The lattice parameters of the bulk Pt crystal were a = b = c = 3.94 Å. Pt was modeled as a (2 × 2) surface unit cell of 4 layers of Pt (111). The lattice parameters of the bulk PtAu crystal were a = 2.80 Å, b = 2.42 Å, and c = 4.84 Å. PtAu was modeled as a (2 × 2) surface unit cell of 4 layers of PtAu (111). The bottom 2 layers were fixed while the others were allowed to relax during the simulation. The Pt and PtAu lattice parameters before and after relaxation was shown in Table 1.

The free energy change (ΔG) was used to evaluate the reaction, which was calculated based on the following equation: ΔG_ads_ = ΔE_ads_ + ΔE_ZPE_ − TΔS, where ΔE_ads_ is the free energy of the pure surface and surface adsorbed molecules, and T is the temperature. ΔE_ZPE_ and ΔS are the zero-point energy change and the entropy change, respectively, which were obtained based on frequency analysis. T is the temperature set to 298.15 K (T = 298.15 K, P = 1 bar). The adsorption molecules of the reaction are *O_2_, *H_2_O, and *HCHO.

## 3. Results and Discussion

### 3.1. Characterization of Morphology and Structure

**Figure 3:**
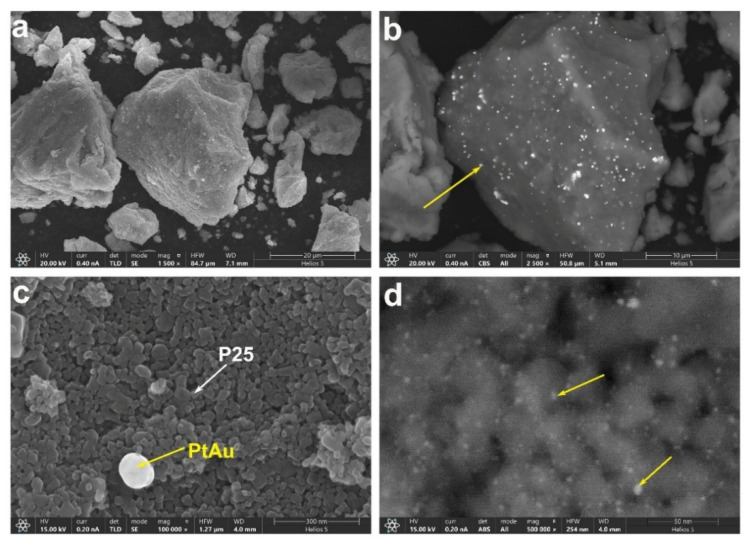
(**a**) Low-magnification secondary electron image of P25(H)-PtAu; (**b**) Low-magnification backscattered electron image of P25 (H)-PtAu; (**c**) Medium-magnification secondary electron image of P25(H)-PtAu; (**d**) High-magnification secondary electron image of P25(H)-PtAu.

Figure 3 presents the SEM images of the P25(H)-PtAu sample. The low-magnification secondary electron imaging reveals that the sample is aggregated into large particles of the micron scale with rough surfaces (Figure 3a). Backscattered electron imaging highlights atomic number-dependent contrast, confirming the homogeneous distribution of high-Z elements across P25 surfaces, identified as Pt/Au (yellow arrow, bright spots in Figure 3a,b). To investigate the microstructure in more detail, a higher magnification image was acquired in Figure 3c. From this SEM image, it is clear that the large aggregates in Figure 3a are composed of P25 (white arrow), with an average particle size of approximately 20–30 nm. As shown in Figure 3d, Nanoscale bright features, measuring 2–5 nm, are observed at ultra-magnification, verifying the successful deposition of PtAu nanoparticles on the P25 substrate. Notably, despite the presence of agglomerates around 100 nm at low magnification (Figure 3c), the majority of PtAu nanoparticles were still nano-sized and uniformly dispersed on the surface of P25, ensuring an adequate distribution of catalytic sites for formaldehyde adsorption and activation.

**Figure 4:**
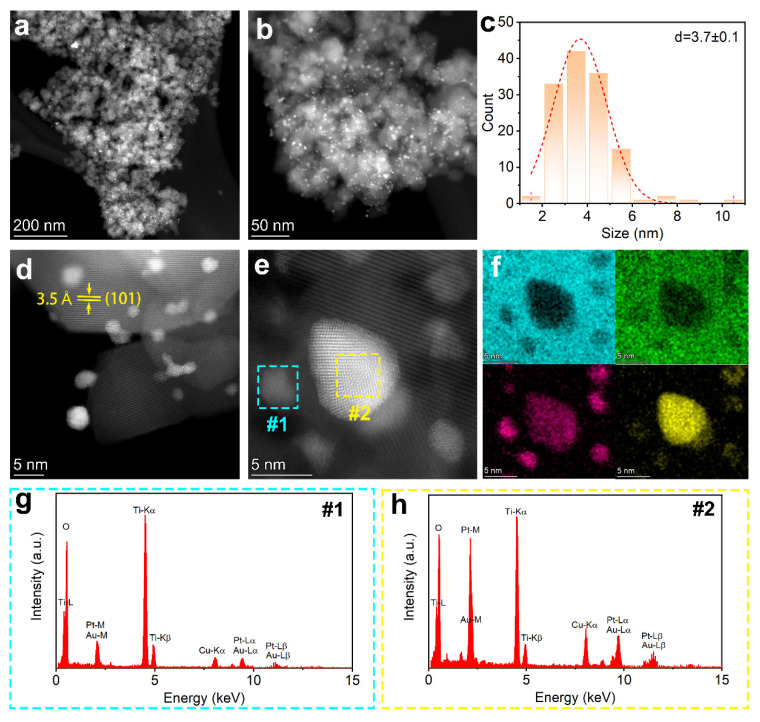
Scanning transmission electron microscopy (STEM) images of P25(H)-PtAu at (**a**) low magnification and (**b**) high magnification; (**c**) The particle size profile of the P25(H)-PtAu; (**d**,**e**) High-resolution STEM images of P25(H)-PtAu; (**f**) EDS elemental maps of Ti, O, Au, and Pt, respectively; (**g**,**h**) The EDX spectra obtained from regions #1 and #2 in (**e**).

Figure 4a,b show the probe-corrected STEM (Z-contrast) images of P25 (H)-PtAu. In these images, the particles exhibiting low contrast are P25, with their size ranging from 20 to 50 nm. On the other hand, the bright spots evenly distributed on the surface of P25 can be attributed to heavy-element clusters (Pt/Au). Those bright nanoparticles feature about 3.7 ± 0.1 nm in size (Figure 4c). This observation is in line with what was seen in the SEM images. As shown in Figure 4d,e, distinct lattice fringes are visible on both the P25 (3.5 Å, Anatase (101)) and PtAu surfaces, indicating the highly crystalline nature of the prepared samples. Figure 4f presents the high-resolution EDS maps of Ti, O, Au, and Pt, respectively. Interestingly, larger nanoparticles (Region #2) exhibit Au-rich PtAu alloys (Figure 4g Au:Pt ≈ 4.3:1), while sub-3 nm clusters (Region #1) show Pt-dominated compositions (Figure 4h Pt:Au ≈ 10:1). The observed dominance of Pt-rich or Au-rich particles in the bimetallic system can be attributed to the thermodynamic immiscibility of Pt and Au. According to the Pt–Au binary phase diagram, these metals exhibit a wide miscibility gap below ~1260 °C, favoring phase separation into Pt-rich (face-centered cubic, FCC) and Au-rich (FCC) domains rather than forming homogeneous alloys [25]. Notably, the nanometer-scale colocalization of Pt and Au signals in elemental mapping demonstrates alloy characteristics, as evidenced by the identical spatial distribution patterns of both elements (Figure 4f).

**Figure 5:**
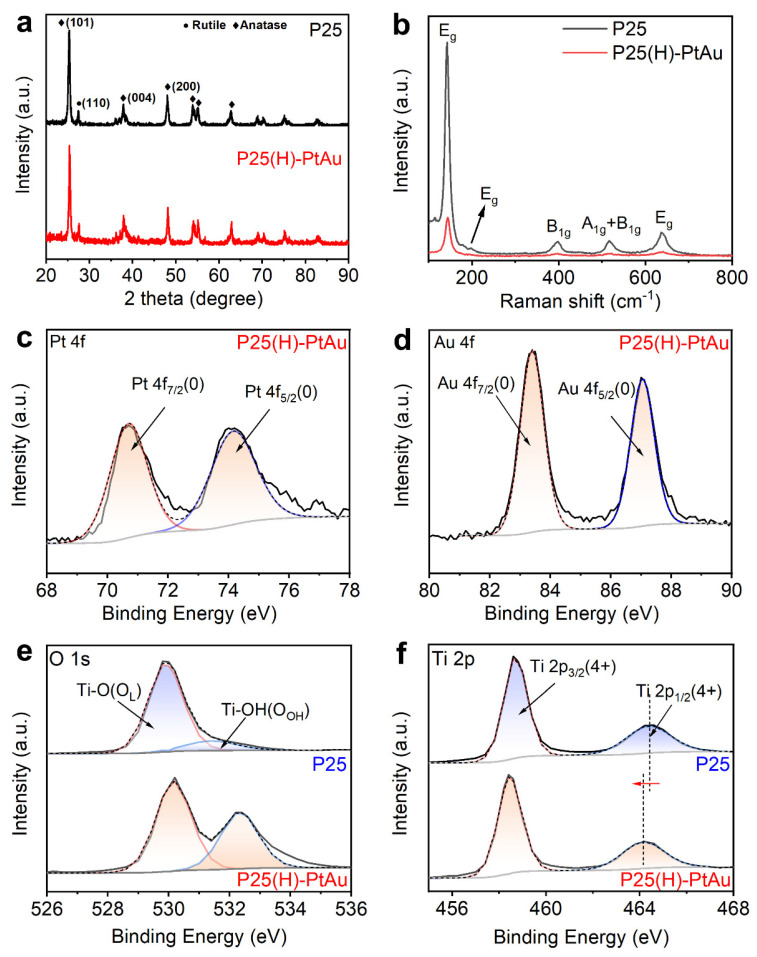
(**a**) XRD and (**b**) Raman patterns for P25 and P25(H)-PtAu; High-resolution XPS spectra of (**c**) Pt 4f, (**d**) Au 4f, (**e**) O 1s, and (**f**) Ti 2p for P25 and P25(H)-PtAu.

As shown in Figure 5a, XRD patterns of pristine P25 and P25(H)-PtAu (Figure 4a) confirm the dominant anatase phase (JCPDS 84-1285) with characteristic peaks at 25.3° (101), 37.8° (004), and 48.0° (200), alongside a minor rutile component (27.4°, 110; JCPDS 86-0148) [26]. Post-synthetic patterns exhibit negligible peak shifts, demonstrating structural preservation of the P25 matrix during hydrogenation and PtAu deposition. At the same time, no distinct Pt or Au reflections were observed in the P25(H)-PtAu sample due to ultrathin nanoparticles below the X-ray diffraction detection limit (Scherrer criteria) [27]. Further evidence of lattice change in TiO_2_ induced by hydrogenation treatment was obtained through Raman spectroscopy. As shown in Figure 5b, pristine P25 demonstrated typical anatase characteristic vibrational modes at 141 cm^−1^ (E_g_), 197 cm^−1^ (E_g_), 397 cm^−1^ (B_1g_), 516 cm^−1^ (A_1g_ + B_1g_), and 636 cm^−1^ (E_g_), where the dominant peak at 141 cm^−1^ corresponds to the O-Ti-O bending vibration mode [28]. Comparative analysis reveals that P25(H)-PtAu shows similar patterns to pristine P25, but these peaks display a decreased intensity, an increased full width at half maximum (FWHM) and a blueshift. These collective spectral changes explicitly indicate that P25 was hydrogenated successfully [29].

The chemical states of the synthesized samples were investigated through high-resolution XPS analysis. As shown in Figure 5c,d, the Pt 4f and Au 4f spectra exhibit binding energies at 70.7 eV (4f_7/2_) and 83.4 eV (4f_7/2_), respectively, confirming the metallic (0) states of both elements. Combined with elemental mapping results, it unequivocally demonstrates the formation of PtAu alloy phases. Figure 5e compares the Ti 2p XPS spectra of P25 and P25(H)-PtAu. It shows characteristic Ti (4+) peaks at 458.6 eV and 464.4 eV, corresponding to the Ti-O-Ti coordination in crystalline TiO_2_. Following hydrogenation, a 0.2 eV negative shift in the Ti 2p is observed, indicating the formation of oxygen vacancies [30]. In addition, O 1s deconvolution peaks of both samples reveal two distinct oxygen species, lattice oxygen (Ti-O-Ti) and hydroxyl groups (Ti-OH). In contrast to P25, the Ti-OH/Ti-O-Ti ratio of P25 (H)-PtAu was significantly increased, directly evidencing hydrogenation-induced replacement of Ti-O-Ti bonds with Ti-OH configurations [31]. Studies have shown that more hydroxyl groups are beneficial to improve the adsorption and photoelectric properties of TiO_2_ in the photocatalytic reaction and guide the transfer of electrons to the surface of TiO_2_ for reaction, thereby improving the photocatalytic performance [32].

### 3.2. Optical Properties

**Figure 6:**
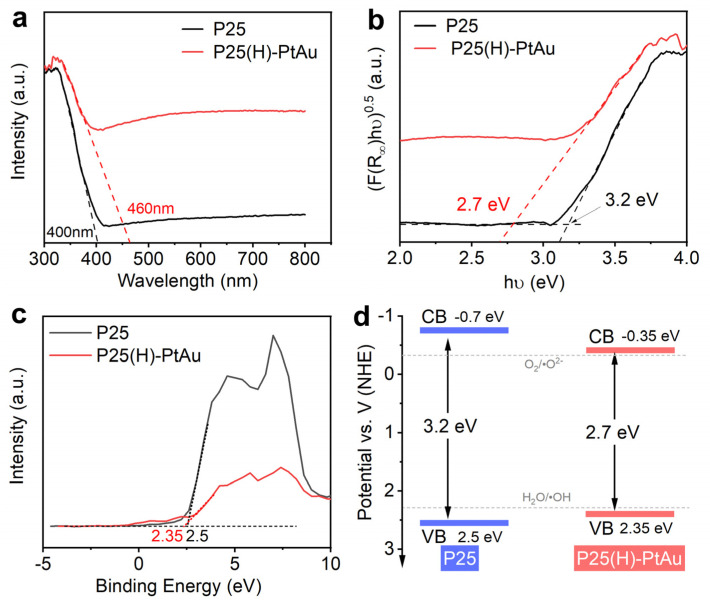
(**a**) UV-vis absorption spectra of P25 and P25(H)-PtAu; (**b**) The plots of transformed K-M function [F(R_∞_)hν]^1/2^ vs. hν for P25 and P25(H)-PtAu; (**c**) The VB spectra of P25 and P25(H)-PtAu; (**d**) The illustration of energy band diagrams for P25 and P25(H)-PtAu.

UV-vis spectroscopy (Figure 6a) reveals a 60 nm red shift in absorption edge (400 → 460 nm) for P25(H)-PtAu compared to pristine P25, accompanied by enhanced visible light harvesting (460–700 nm). Kubelka–Munk analysis (Figure 6b) quantifies the bandgap (E_g_) reduction from 3.2 eV (P25) to 2.7 eV (P25(H)-PtAu) [33]. As shown in Figure 6c, PristineP25 exhibits a valence band maximum (VBM) at 2.0 eV, whereas the P25(H)-PtAu sample demonstrates an upward-shifted VBM at 2.35 eV. Based on the above energy level information and the fundamental semiconductor relationship E_CBM_ = E_VBM_ − E_g_, the conductive band minimum (CBM) of P25 and P25(H)-PtAu was calculated in −0.7 eV and −0.35 eV. Thus, the band alignments of P25 and P25(H)-PtAu were systematically constructed, as presented in Figure 6d. It is noted that P25(H)-PtAu retains thermodynamic compatibility with redox potentials for photocatalytic reactions (H_2_O/•OH at +2.31 V vs. NHE; O_2_/•O_2_^–^ at −0.33 V vs. NHE) after hydrogenation [34]. On the whole, this delicate balance ensures that the hydrogenated P25(H)-PtAu preserves its ability to generate reactive oxygen species while expanding light absorption into the visible spectrum.

### 3.3. Photocatalytic Performance

**Figure 7:**
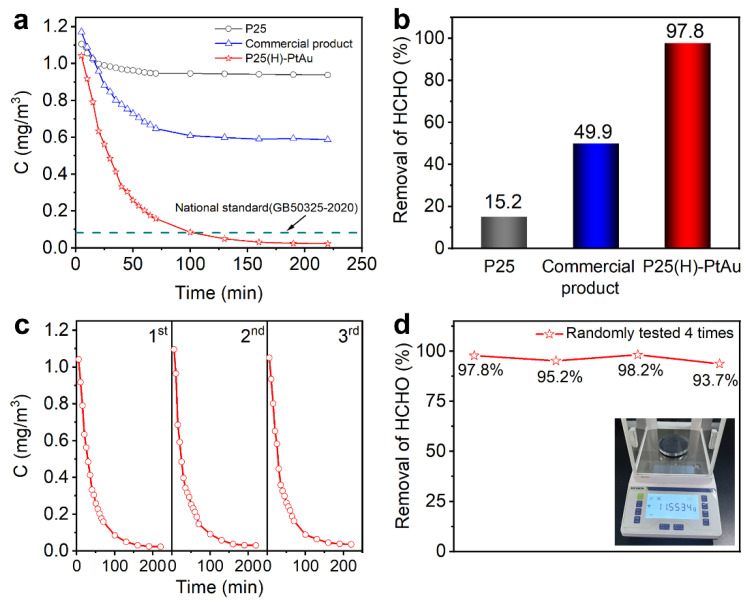
(**a**) The concentration of formaldehyde at room temperature as a function of reaction time over pristine P25, a commercial product, and P25(H)-PtAu; (**b**) The efficiency of formaldehyde removal using different photocatalysts; (**c**) The recycling test of formaldehyde removal for P25(H)-PtAu; (**d**) Four random sampling tests in a batch of 10 g for P25(H)-PtAu.

Figure 7a illustrates the formaldehyde concentration at room temperature as a function of reaction time over pristine P25, a commercial product, and P25(H)-PtAu. Initially, the formaldehyde concentration was controlled in the range of 1.0–1.2 mg/m^3^. Under the influence of the three catalysts, the formaldehyde concentration decreased rapidly, though the rate of decrease slowed down after 30 min. By 70 min, the concentrations of formaldehyde treated with P25 and the commercial catalyst stabilized at 0.95 mg/m^3^ and 0.61 mg/m^3^, respectively, still significantly higher than the national standard of 0.08 mg/m^3^. In contrast, the sample prepared in our study achieved a formaldehyde concentration of only 0.085 mg/m^3^ after 100 min of light irradiation, which is slightly above the national standard. With continued exposure, the concentration further decreased to 0.023 mg/m^3^, effectively reaching a safe level. Eventually, the removal efficiency of formaldehyde for P25(H)-PtAu was up to 97.8%, much higher than that of pristine P25 (15.2%) and a commercial product (49.9%) (as shown in Figure 7b). To further evaluate the photocatalytic stability of the designed catalysts, the photocatalytic stability of the P25(H)-PtAu for FA degradation was examined over three cycles in Figure 7c. The experimental results showed good stability in the photocatalytic performance of FA degradation over the three reuse cycles without significant change. The P25(H)-PtAu catalyst developed in this work demonstrates significant advantages in both efficiency and stability compared to other reported oxide-type catalysts (Table 2). We further increased the production scale to manufacture a single batch of 10 g of the product. Subsequently, four random sampling tests were conducted on these products. It can be seen that the change in formaldehyde degradation performance of the above products is <5% (Figure 7d), which indicates that our method establishes a feasible pathway for industrial-scale air purification applications.

### 3.4. Mechanism Analysis

**Figure 8:**
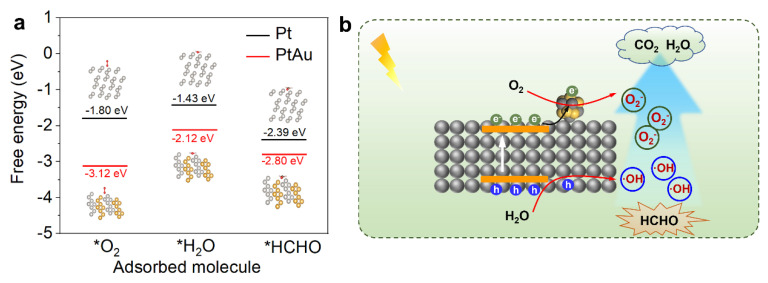
(**a**) Free energy diagram of adsorbed reactants (O_2_, H_2_O, and HCHO) for Pt and PtAu; (**b**) Plausible mechanisms of photocatalytic degradation of formaldehyde over P25(H)-PtAu.

Density functional theory (DFT) calculations were performed to elucidate the adsorption behavior of reactants (H_2_O, O_2_, and FA) on Pt and PtAu alloy surfaces. As shown in Figure 8a, all adsorbed reactants exhibit lower (more negative) free energy values on PtAu alloy surfaces than on monometallic Pt, which indicates PtAu has a stronger adsorption effect on reactants, and thus can improve surface reaction kinetics in formaldehyde degradation. Combined with the above analysis, the improved performance of our samples can be attributed to several factors: Firstly, the hydrogenation treatment enhances the visible light absorption of the material. This expansion in the absorption range allows the catalyst to utilize a broader spectrum of light, which is crucial for driving photocatalytic reactions. Secondly, the incorporation of PtAu noble metals introduces additional active sites on the catalyst, facilitating better adsorption and transformation of reactants, leading to improved catalytic performance. Additionally, PtAu also enhances the separation of charge carriers generated during the photocatalytic process and utilization of charge carriers generated during the photocatalytic process, further boosting efficiency in removing formaldehyde.

Based on the above experimental and computational results, we propose a possible FA degradation mechanism. As shown in Figure 8b, under light irradiation, P25 generates electron-hole pairs (e^−^-h^+^), where the photogenerated electrons are rapidly captured by PtAu nanoalloys via Schottky junction-mediated electron transfer, effectively suppressing charge recombination. At the same time, the holes migrate to the P25 surface, oxidizing surface hydroxyl groups (-OH) to yield highly reactive hydroxyl radicals (•OH). The oxygen reduction reaction (O_2_ + e^−^ → •O_2_^−^) on PtAu surfaces further generates superoxide radicals, synergizing with •OH to establish a multi-radical oxidation system. Under the action of •OH and •O_2_^−^, formaldehyde molecules can be degraded to CO_2_ and H_2_O molecules, thus achieving air purification.

## 4. Conclusions

Herein, we propose a scalable two-step strategy for the synthesis of high-performance P25(H)-PtAu photocatalysts. Hydrogenation-induced rich defects enhance visible light absorption of P25 and improve light utilization. The ultrasmall PtAu nanoalloys effectively enhance charge separation while optimizing reactant adsorption, thus enhancing surface reaction kinetics. The prepared P25(H)-PtAu catalyst shows much better efficiency (97.8%) than the currently available photocatalysts in the commercial market. Within 2 h, the formaldehyde concentration can be reduced to a safe level even in a sealed environment. The experimental results further demonstrated excellent stability and high reproducibility in the photocatalytic degradation of FA. What is even more significant is that this method enables the preparation of batches, specifically 10 g batches. This scalability is a crucial step towards industrial production and wider implementation of the photocatalytic system for effective formaldehyde removal.

## Figures and Tables

**Table 1:** Pt and PtAu lattice parameters before and after relaxation.

Unit Cell	Lattice Parameters		
Pt-initial	a = 3.94 Å	b = 3.94 Å	c = 3.94 Å
Pt-relaxation	a = 3.98 Å	b = 3.98 Å	c = 3.98 Å
PtAu-initial	a = 2.80 Å	b = 2.42 Å	c = 4.84 Å
PtAu-relaxation	a = 2.82 Å	b = 2.44 Å	c =4.93 Å

**Table 2:** Compared the performances of P25(H)-PtAu with other reported literature.

Materials	Light Source	Efficiency (%)	Time (min)	Ref.
P25(H)-PtAu	300 W Xe lamp 50 mW/cm^2^	97.8	240	This work
Nano-ZnO	Ultraviolet intensity 1180 mW/cm^2^	73	40	[35]
Bi_2_O_3_/TiO_2_	36 W LED	94	24 h	[36]
rGO/TiO_2_	500 W Xe lamp	88.3	240	[37]
Bi_2_MoO_6_/Bi/g-C_3_N_4_	350 W Xe lamp (λ > 420 nm)	96.15	10 h	[38]
Cu-TiO_2_	500 W Xe lamp	100	140	[39]
TiO_2_ nanotube	Two low-pressure mercury lamps	94	75	[40]
BFO@OCN	ultraviolet light (365 nm)	55	90	[41]
K-C_3_N_4_/Ag/Ag_3_PMo_12_O_40_	(λ > 420 nm)	90	60	[42]
Bi_2_MoO_6_	Visible light	94	180	[43]
BiOI@Carbon	Visible light	73	40	[44]

## Data Availability

Data is contained within the article.
